# Extracellular Adenosine Protects against *Streptococcus pneumoniae* Lung Infection by Regulating Pulmonary Neutrophil Recruitment

**DOI:** 10.1371/journal.ppat.1005126

**Published:** 2015-08-27

**Authors:** Elsa N. Bou Ghanem, Stacie Clark, Sara E. Roggensack, Sally R. McIver, Pilar Alcaide, Philip G. Haydon, John M. Leong

**Affiliations:** 1 Department of Molecular Biology and Microbiology, Tufts University School of Medicine, Boston, Massachusetts, United States of America; 2 Program in Molecular Microbiology, Sackler School of Graduate Biomedical Sciences, Tufts University, Boston, Massachusetts, United States of America; 3 Department of Neuroscience, Tufts University School of Medicine, Boston, Massachusetts, United States of America; 4 Sackler School of Graduate Biomedical Sciences, Tufts University School of Medicine and Molecular Cardiology Research Institute, Tufts Medical Center, Boston, Massachusetts, United States of America; University of Birmingham, UNITED KINGDOM

## Abstract

An important determinant of disease following *Streptococcus pneumoniae* (pneumococcus) lung infection is pulmonary inflammation mediated by polymorphonuclear leukocytes (PMNs). We found that upon intratracheal challenge of mice, recruitment of PMNs into the lungs within the first 3 hours coincided with decreased pulmonary pneumococci, whereas large numbers of pulmonary PMNs beyond 12 hours correlated with a greater bacterial burden. Indeed, mice that survived infection largely resolved inflammation by 72 hours, and PMN depletion at peak infiltration, i.e. 18 hours post-infection, lowered bacterial numbers and enhanced survival. We investigated host signaling pathways that influence both pneumococcus clearance and pulmonary inflammation. Pharmacologic inhibition and/or genetic ablation of enzymes that generate extracellular adenosine (EAD) (e.g. the ectoenzyme CD73) or degrade EAD (e.g. adenosine deaminase) revealed that EAD dramatically increases murine resistance to *S*. *pneumoniae* lung infection. Moreover, adenosine diminished PMN movement across endothelial monolayers *in vitro*, and although inhibition or deficiency of CD73 had no discernible impact on PMN recruitment within the first 6 hours after intratracheal inoculation of mice, these measures enhanced PMN numbers in the pulmonary interstitium after 18 hours of infection, culminating in dramatically elevated numbers of pulmonary PMNs at three days post-infection. When assessed at this time point, *CD73* ^*-/-*^ mice displayed increased levels of cellular factors that promote leukocyte migration, such as CXCL2 chemokine in the murine lung, as well as CXCR2 and β-2 integrin on the surface of pulmonary PMNs. The enhanced pneumococcal susceptibility of *CD73* ^*-/-*^ mice was significantly reversed by PMN depletion following infection, suggesting that EAD-mediated resistance is largely mediated by its effects on PMNs. Finally, CD73-inhibition diminished the ability of PMNs to kill pneumococci *in vitro*, suggesting that EAD alters both the recruitment and bacteriocidal function of PMNs. The EAD-pathway may provide a therapeutic target for regulating potentially harmful inflammatory host responses during Gram-positive bacterial pneumonia.

## Author Summary

Despite the presence of vaccines and antibiotic therapies, invasive *Streptococcus pneumoniae* (pneumococcus) infections, such as pneumonia, bacteremia and meningitis, remain a leading cause of mortality and morbidity worldwide. Understanding the host factors that influence the outcome of *S*. *pneumoniae* infection will allow us to design better therapies. Here, we elucidate the role of rapidly responding innate immune cells termed neutrophils, or PMNs (polymorphonuclear leukocytes), whose role in *S*. *pneumoniae* infection has long been controversial. We found that PMNs are initially required for controlling bacterial numbers, but their extended presence in the lungs leads to significant damage and poor control of infection. The signals that control the movement of PMNs into the infected lungs are not well understood. Here, we identified extracellular adenosine (EAD), a molecule produced by the host in response to cellular damage, as important in limiting PMN movement into the lungs upon pneumococcal challenge. Importantly, EAD-mediated control of PMNs was crucial for fighting lung infection by *S*. *pneumoniae*. This study may lead to the potential use of clinically available adenosine-based therapies to combat pneumococcal pneumonia in the future.

## Introduction

Despite vaccines and antibiotic therapies, invasive *Streptococcus pneumoniae* (pneumococcus) infections such as pneumonia, meningitis and bacteremia remain a considerable health and economic burden [1,2]. A major determinant of disease following *S*. *pneumoniae* infection is pulmonary inflammation, which, if excessive, can result in tissue destruction, compromised gas exchange, and/or acute respiratory distress syndrome [3]. Many conditions associated with enhanced inflammation, including influenza infection [4–6] and aging [7,8], lead to increased susceptibility to pneumococcal pneumonia.

Effective inflammatory responses to infection balance host defense with the potentially competing demand of a rapid return to homeostasis. Indeed, pneumococcal pneumonia triggers a massive neutrophil, or polymorphonuclear leukocyte (PMN), influx into the alveolar spaces [9,10], but the role of these innate immune cells during infection is complex. Several findings suggest that PMNs are needed to control the infection: neutropenic patients are at increased risk for pneumonia [11], and in several mouse studies, depletion of PMNs prior to *S*. *pneumoniae* infection [12,13] or delay in PMN recruitment into the lungs [14,15] resulted in higher pulmonary bacterial loads and lethal septicemia. Paradoxically however, conditions associated with increased numbers of PMNs in the lungs several days after *S*. *pneumoniae* infection of mice, such as advanced age [8,16], deficiency in regulatory T cells [17], or influenza infection [18], result in more severe systemic infection and reduced survival. Conversely, reducing PMN influx into mouse airways dramatically decreases bacteremia, resulting in uniform survival to a normally lethal pneumococcal pulmonary challenge [9]. These findings suggest that host survival may require an initial acute PMN response that is rapidly resolved later in the course of *S*. *pneumoniae* infection.

To reach *S*. *pneumoniae* in alveolar spaces, circulating PMNs cross the endothelium, enter into the interstitial space, then breach the lung epithelium to access the airway spaces [19]. This complex process involves multiple pathways of chemotaxis, including those mediated by eicosanoids [9] or chemokines [19] [20], as well as a network of ligand-receptor interactions, including those mediated by lectins or integrins [15]. Although many studies have focused on positive regulators of PMN recruitment into the lungs following pneumococcal challenge [9,14,15], signals that negatively regulate this process and ultimately promote resolution of this response are poorly understood.

Extracellular adenosine (EAD) is a potentially crucial regulator of PMN-mediated pulmonary inflammation. Basal EAD levels in tissues are typically low (<1μM) [21], but can increase more than ten-fold during pathological conditions [22]. Upon cellular insult, such as infection [23], ATP is released from cells and metabolized to adenosine by the sequential action of two extracellular enzymes, CD39, which converts ATP to AMP, and CD73, an ecto-5’-nucleotidase that de-phosphorylates AMP to EAD [22]. EAD is recognized by four G-protein coupled receptors, A1, A2A, A2B and A3 [23] leading to enhanced or diminished acute inflammation, depending on the target receptor, cell type, and/or EAD concentration [23]. Thus, the EAD pathway may provide a means for complex regulation of PMN movement [22].

Several non-infectious acute pulmonary injury models indicate that EAD generated by endothelial cell CD73 binds to cognate adenosine receptors on PMNs, leading to reduced PMN-endothelial cell adhesion, inflammation, and tissue damage [24–26]. Lung epithelial cells are both an important EAD source [25] and, given that they produce all four adenosine receptors [21], a potential EAD target. *CD73* ^*-/-*^ mice show impaired clearance of bacteremia and enhanced pulmonary inflammation in a cecal puncture model [27], whereas deficiency of adenosine A2B or A1 receptors was protective against *Klebsiella pneumoniae* [28] or influenza lung infection [29], respectively. Thus, the role of EAD in pathogen lung burden, inflammation, and injury during bacterial infection is not fully characterized.

In this study, we characterized the kinetics of PMN entry into the lung during murine pneumococcal challenge with an invasive *S*. *pneumoniae* strain, and addressed potential beneficial and detrimental roles of PMNs in disease. We found that PMNs promoted microbial control early, but inhibited bacterial clearance later in infection. We identified the EAD pathway as a regulator of endothelial transmigration and PMN recruitment into the lung at later time points after pneumococcal infection, as well as PMNs anti- pneumococci function. This study is a first step in elucidating the potentially complex role of the EAD-pathway in regulating pulmonary inflammation and host defense against Gram-positive bacterial pneumonia.

## Results

### Kinetic analysis of PMN influx into the lungs and bacterial infection following *S*. *pneumoniae* intratracheal challenge

**Fig 1:**
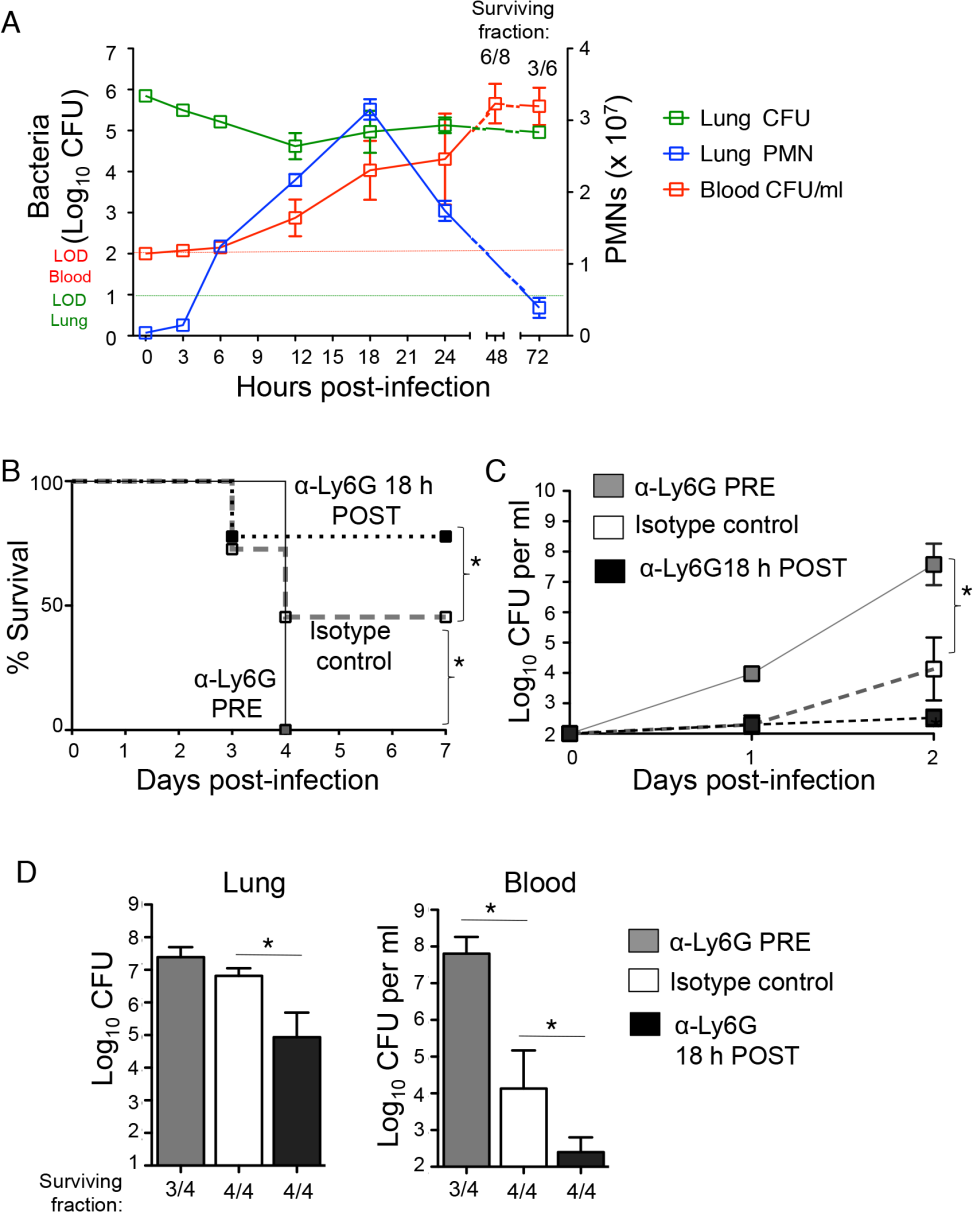
PMNs promote pulmonary and systemic disease during later stages of *S*. *pneumoniae* lung infection. (A) C57BL/6J mice were inoculated I.T with 5x10^5^ CFU of *S*. *pneumoniae* TIGR4 and pulmonary (green) and bloodstream (red) bacterial loads, as well as pulmonary PMNs (blue) were monitored through 72 hours post-infection. Shown are representative data from one of two separate experiments (using 3 to 4 mice per time point). The numbers above the graph represent the fraction of surviving mice within that group at the corresponding time. (B-D) C57BL/6J mice were treated i.p with PMN depleting antibodies (anti-ly6G) or isotype control either 18 hours pre or post pulmonary challenge with 5x10^5^ CFU of *S*. *pneumoniae* TIGR4. Survival (B) and bacterial burdens in the blood (C) were monitored over time and shown are pooled data from two separate experiments. (D) Pneumococcal burdens in the lungs and blood were determined 3 days post-infection. The numbers below the graph represent the fraction of surviving mice within that group. Representative data from one of 4 separate experiments with 3 to 4 mice per group are shown. Means +/- SEM are given in Panels A, C and D, and values significantly (*p*<0.05) different from isotype control-treated group by Student’s t-test are indicated by asterisk. In Panel B, asterisk indicates survival rate was significantly (*p*<0.05) different from isotype control-treated controls by Log-rank (Mantel-Cox) test.

To better understand the role of PMNs following pneumococcal infection, C57BL/6 mice were infected intra-tracheally (I.T.) with 5x10^5^ colony-forming units (CFU) of *S*. *pneumoniae* TIGR4 strain and pulmonary PMN influx and bacterial burdens in the lungs and blood were monitored for 72 hours. The total number of pulmonary PMNs, determined by flow cytometric analysis of a single-cell suspension of whole lung, increased four-fold in the first three hours post-infection, then underwent a dramatic increase, peaking at 30 million, i.e. ~100-fold greater than uninfected controls, at 18 hours post-infection (Fig 1A). Between 24 and 72 hours post-infection, as mice started to succumb to the disease, surviving mice experienced an ~10-fold decrease in pulmonary PMNs (Fig 1A).

Quantitation of bacterial numbers in the lung revealed two phases of infection control. In spite of the fact that *S*. *pneumoniae* TIGR4 is a virulent strain capable of replication in the murine lung [30], bacterial numbers in the lung decreased ~30-fold in the first 12 hours of infection, a period in which PMN numbers increased dramatically (Fig 1A). However, between 12 and 18 hours, during which mice continued to experience a striking increase in pulmonary PMNs, bacterial lung burden increased 5-fold to approximately 2 x 10^5^, and this level of infection or higher was maintained for the remainder of the 72-hour experiment. Moreover, the three-fold increase in pulmonary PMNs, peaking at 18 hours post-challenge correlated with a large increase in bacterial numbers in the circulation, with titers of more than 10^4^/ml, consistent with our previous findings that PMN entry into the lung facilitates bacterial spread [9]. Over the next 30 hours, a majority of infected mice succumbed to infection (Fig 1A) and even among survivors, bacterial titers in the blood increased 100-fold to over a million CFU/ml. Thus, although the initial increase in PMN influx into the lungs corresponded to a transient control of infection during the first 12 hours, the further accumulation of PMNs after this time point, peaking at 18 hours post-infection, coincided with the development of serious systemic infection.

### PMNs are required for protection at the beginning of infection, but are detrimental at later times

To experimentally address the role of PMNs during lung infection by *S*. *pneumoniae*, we depleted PMNs with intra-peritoneal (i.p.) injections of the anti-Ly6G antibody (IA8) either one day before I.T. infection with ~5x10^5^ colony forming units (CFU) of *S*. *pneumoniae* TIGR4 strain, or 18 hours post-infection (see Methods), a time point that corresponded to peak pulmonary infiltration by PMNs (Fig 1A). At both time points, treatment with the anti-Ly6G antibody resulted in >90% depletion of lung and circulating PMNs compared to isotype-treated controls (see Methods). Survival and bacteremia, as well bacterial burdens in the lungs and blood at day three following infection were compared between the groups (Fig 1). Consistent with previous reports [12,13], mice depleted of PMNs pre-infection were extremely susceptible to *S*. *pneumoniae* (Fig 1B). In comparison to the matched isotype-treated control group, the pre-depleted mice suffered more than a thousand-fold greater bacterial load in the bloodstream (Fig 1C and 1D), and failed to survive the infection (Fig 1B).

In contrast, depletion of PMNs at 18 hours post-infection significantly increased the survival rate (Fig 1B) and lowered bacterial burdens a hundred-fold in both the lungs and blood (Fig 1C and 1D). Our findings strongly support the hypothesis that while PMNs are required for bacterial control at the beginning of pneumococcal infection, their persistence following infection is detrimental to the host.

### Inhibition of adenosine breakdown promotes host defense against *S*. *pneumoniae*

**Fig 2:**
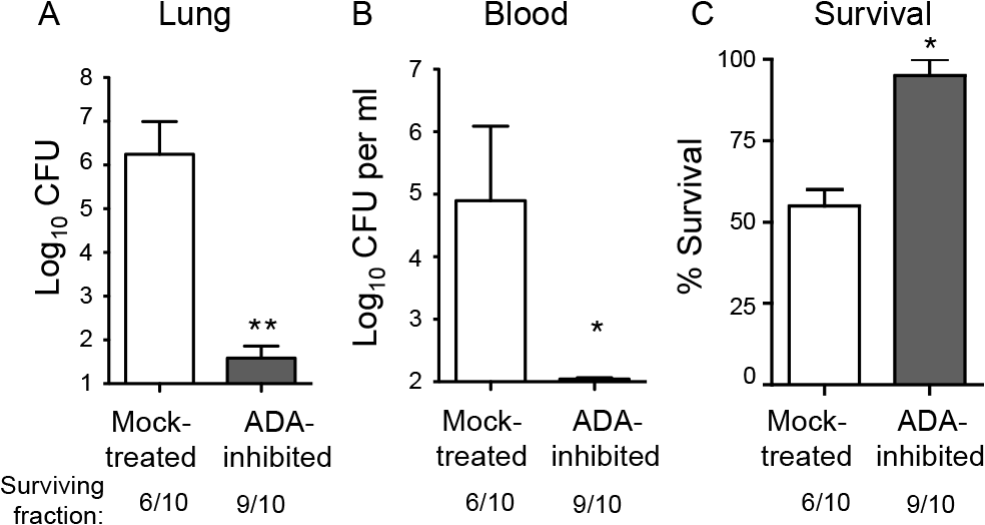
Inhibition of adenosine breakdown promotes resistance to *S*. *pneumoniae* lung challenge. C57BL/6J mice mock-treated or treated with EHNA-hydrochloride, an adenosine deaminase inhibitor, were inoculated I.T with 5x10^5^ CFU of *S*. *pneumoniae* TIGR4. Bacterial burdens in the lungs (A) or blood (B), as well as survival (C), was determined 3 days post-infection. Data pooled from 2 separate experiments (n = 6 mice per group) are shown. Data represent means +/- SEM. ** = *p*< 0.001 and * = *p*<0.05 indicate means significantly different from mock-treated group by Student’s t-test. Below the graphs are indicated the fraction of surviving mice within each group.

A potentially crucial regulator of PMN-mediated pulmonary inflammation is extracellular adenosine (EAD; [23]). To test its role in resistance against serious infection by *S*. *pneumoniae*, we first inhibited adenosine deaminase (ADA), an enzyme responsible for the breakdown of adenosine [31]. Mice were subjected to i.p. injections of EHNA hydrochloride, a pharmacological inhibitor of ADA [32] that was previously shown to increase EAD levels in mice [33]. The mice were then challenged I.T. with 5x10^5^ CFU, a normally lethal dose of *S*. *pneumoniae*, and bacterial burdens in the lung and blood were determined three days post-infection. Whereas mock-treated mice suffered high levels of bacteria in the lungs and blood, ADA-inhibited mice had on average ten thousand-fold fewer pneumococci in the lungs (Fig 2A) and were free of detectable bacteremia (Fig 2B). By day 3 post-infection, 40% of the mock-treated mice had succumbed to the infection, compared to 10% of the ADA-inhibited mice (Fig 2C). Although in some infection models, adenosine-mediated protection is due to the direct effects of adenosine on the infectious agent [34], we found that adenosine concentrations typically present in inflamed tissues [22] had no effect on bacterial growth *in vitro* (S1 Fig).

Since the ADA inhibitor EHNA hydrochloride may also target other enzymes [35], we tested whether the protective phenotype was dependent on adenosine signaling. Adenosine receptor blockade partially reversed the protective effect of inhibition of adenosine breakdown by ADA (S2 Fig), consistent with the hypothesis that the protection we observed upon treatment with EHNA hydrochloride is at least partly mediated via the interaction between adenosine and its receptors.

### Inhibition of CD73 increases susceptibility of mice to infection after *S*. *pneumoniae* lung challenge

**Fig 3:**
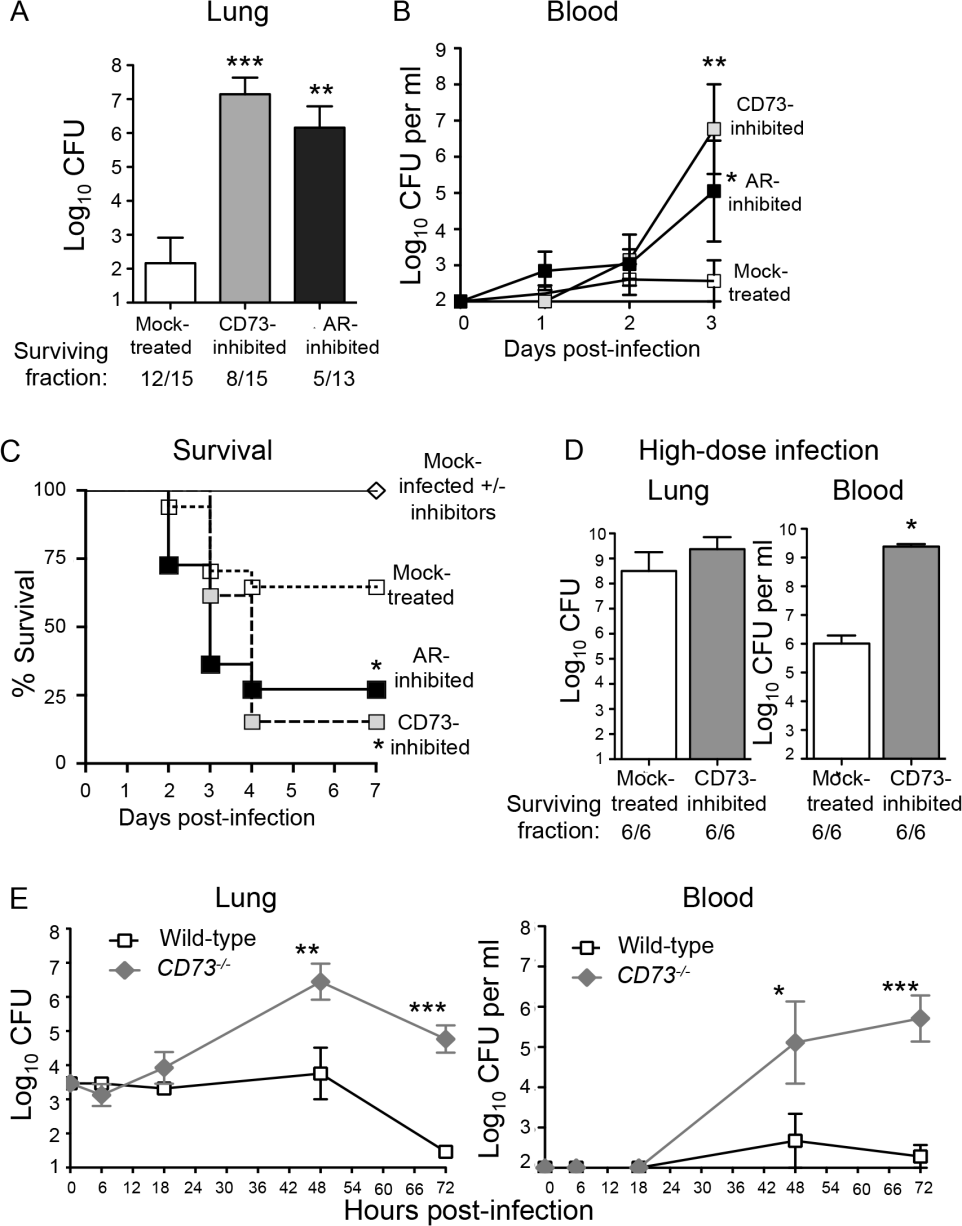
Inhibition of EAD production or signaling enhances susceptibility of mice to *S*. *pneumoniae* lung challenge. (A-D) Wild-type C57BL/6J mice were either treated with the CD73 inhibitor (α,β- methylene ADP), the pan adenosine receptor (AR) inhibitor (CGS 15943) or mock-treated with a vehicle control. (A) Bacterial loads in the lungs of a group of mice were determined 3 days after I.T. inoculation with 5x10^3^ CFU of *S*. *pneumoniae* TIGR4. (B-C) Bacteremia (B) and survival (C) were monitored over time for another group of mice. Pooled data from 3 separate experiments (n = 6–12 mice per group) are shown. (D) Mice were challenged I.T. with a high dose, ~1x10^7^ CFU of *S*. *pneumoniae* TIGR4 and bacterial burdens in the lungs and blood were assessed at two days post-infection. Pooled data from two separate experiments (n = 6 mice per group) are shown. Below figures A and D are indicated the fraction of surviving mice within each group. (E) *CD73* ^*-/-*^ mice and wild-type B6 controls were inoculated I.T. with 5x10^3^ CFU of *S*. *pneumoniae* TIGR4 and bacterial burdens in the lungs and blood were measured at the indicated time points post-infection. Data pooled from 3 separate experiments (n = 6–9 mice per group) are shown. None of the mice died within the time frame of these (E) experiments. Data represent means +/- SEM. Means that are significantly different from mock-treated group (A-B) or wild-type control group (E) by student t-test are indicated by asterisks (*** = *p*< 0.0001; ** = *p*< 0.001; * = *p*<0.05). In Panel C, a survival rate significantly (*p*<0.05) different from mock-treated controls by Log-rank (Mantel-Cox) test is indicated by asterisk.

A prediction of the hypothesis that EAD is responsible for the protective effect of ADA inhibition is that inhibition of adenosine production should enhance susceptibility to infection. To test that, wild-type C57BL/6 mice were either mock-treated or injected intra-peritoneally with the CD73 inhibitor α,β methylene ADP which was shown to drastically lower adenosine levels in mice [24]. The mice were then challenged I.T. with 5x10^3^ *S*. *pneumoniae* TIGR4, a dose ~2-fold below the LD_50_. Neither the addition of adenosine or α,β methylene ADP had any effect on *S*. *pneumoniae* growth *in vitro* (S1 Fig). However, at day 3 post-infection, mice treated with the inhibitor suffered approximately a million-fold higher bacterial burden in their lungs compared to mock-treated controls (Fig 3A). In addition, whereas mock-treated mice suffered low-level bacteremia that eventually resolved, CD73-inhibited mice suffered bacteremia that reached a million CFU per milliliter of blood at day 3 post-infection (Fig 3B). To determine if CD73 inhibition resulted in enhanced bacteremia in a mouse strain more resistant to *S*. *pneumoniae* infection [36], we I.T. challenged either mock-treated or CD73-inhibited BALB/c mice and found that CD73 inhibition was associated with a 100- to 1000-fold increase in bacteremia after day 3 post-infection (S3 Fig). In C57BL/6 mice, CD73 inhibition was also associated with apparent neurological dysfunction, such as hind limb twitching, weakness, paralysis and an inability to walk normally. By day 4 post-infection, 85% of CD73-inhibited C57BL/6 mice succumbed to the infection (Fig 3C).

To test whether the increased systemic spread of pneumococci was simply a reflection of increased bacterial loads in the lungs, we challenged mice with a high dose of *S*. *pneumoniae*, i.e. 2x10^7^ CFU. CD73-inhibited mice suffered only a 1.1-fold higher (and statistically indistinguishable) bacterial lung burden than mock-treated mice (Fig 3D). Despite similar numbers of bacteria in the lung, CD73-inhibition resulted in 1000-fold higher levels of bacteremia. Our findings suggest that in addition to impacting the ability of the host to control lung infection, CD73 inhibition also promotes systemic spread of *S*. *pneumoniae* from the lungs.

To test the role of CD73 during pneumococcal infection using genetic rather than pharmacological means, and to determine whether CD73 activity alters bacterial load early in infection, we inoculated *CD73* ^*-/-*^ mice I.T with 5x 10^3^ *S*. *pneumoniae* and followed lung and blood CFU over time. CD73-deficiency had no significant effect on bacterial burden at either site at 6 or 18 hours post-infection (Fig 3E), suggesting that EAD does not play a major role in controlling bacterial numbers at the early stages of infection. Beyond 18 hours post-infection, *CD73* ^-/-^ mice were incapable of controlling pneumococcal burdens, reflected in a 100- to 1000-fold increase in both infection sites (Fig 3E). In contrast, bacterial numbers in the lung and blood of wild-type mice increased only slightly in the first 48 hours of infection and were largely cleared by 72 hours. Thus, pharmacological inhibition or genetic ablation of CD73, an enzyme required for EAD production [24], drastically increased the *S*. *pneumoniae* lung burden and susceptibility to systemic disease.

### The effect of EAD on host susceptibility to pneumococcal challenge is dependent on adenosine receptor signaling

To test whether EAD-mediated protection upon pneumococcal infection was dependent on signaling via adenosine receptors in the host, mice were treated with the pan-adenosine receptor antagonist CGS-15943 [37] prior to challenge with *S*. *pneumoniae*. This inhibitor targets all four adenosine receptors, with Ki values of 3.5, 4.2, 16 and 51 nM for human A1, A2A, A2B and A3 receptors respectively [37,38]. Although CGS-15943 had no effect on the viability of *S*. *pneumoniae in vitro* (S1 Fig), treatment of mice with this inhibitor resulted in increased susceptibility to *S*. *pneumoniae* lung challenge that was virtually identical to that observed upon inhibition of CD73-mediated EAD production (Fig 3). Compared to mock-treated controls, mice treated with the adenosine receptors antagonist suffered ten thousand-fold higher bacterial loads in their lungs (Fig 3A) as well as bacteremia exceeding 10^3^ CFU/ml (Fig 3B). The mice treated with the adenosine receptors antagonist also displayed a significant survival defect compared to mock-treated mice following pneumococcal lung challenge (Fig 3C). These findings clearly show that inhibition of adenosine receptors signaling render mice highly susceptible to pneumococcal challenge.

### Extracellular adenosine signaling diminishes recruitment of PMNs across endothelial but not epithelial monolayers

**Fig 4:**
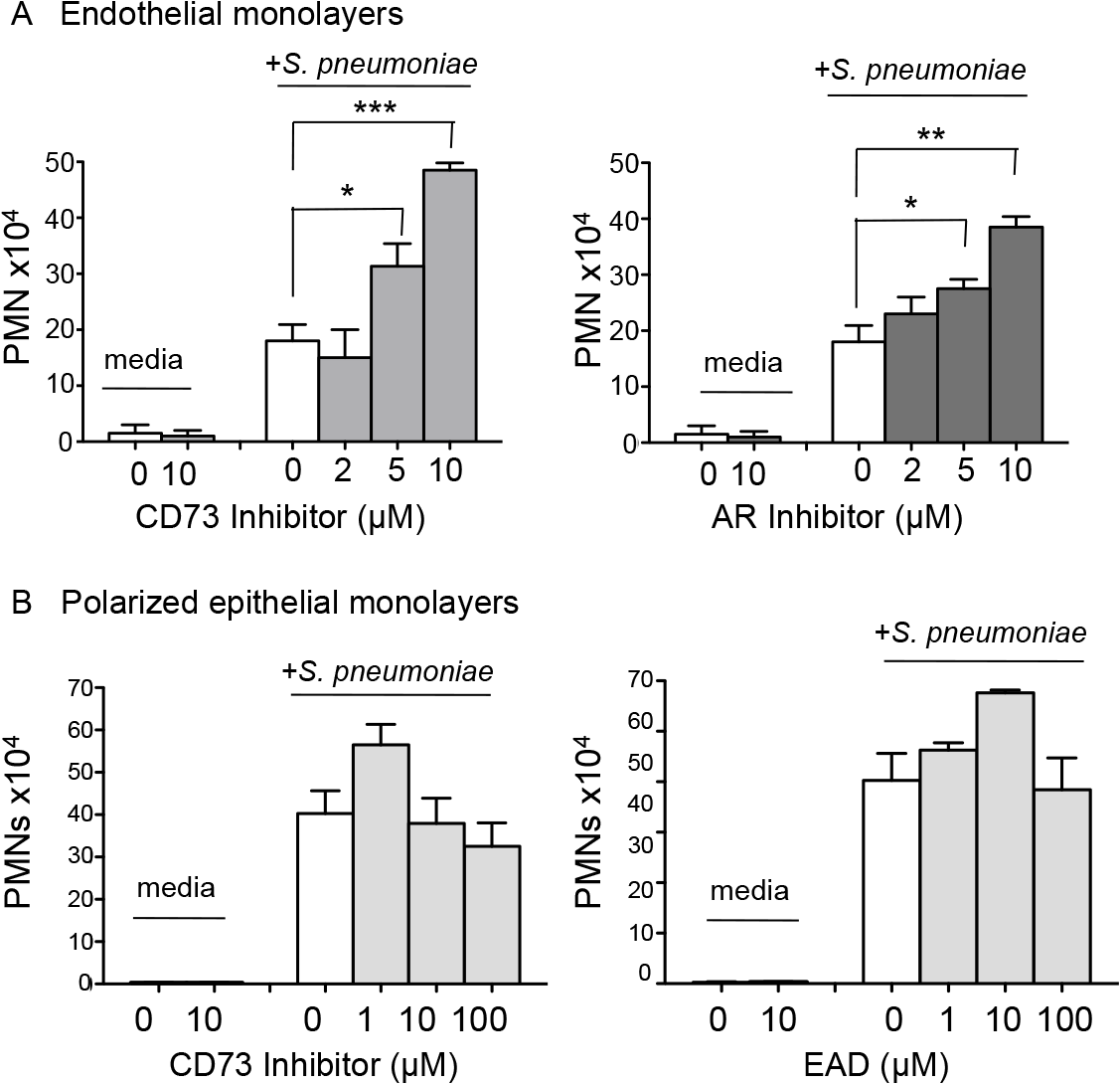
EAD negatively regulates PMN migration across the endothelium but not the epithelium. (A) Media alone or *S*. *pneumoniae-*containing media was added to the lower chamber of HUVEC-seeded Transwell dishes for 3 hours. Transmigration of PMNs added to the upper chamber in media containing vehicle control or increasing concentrations of the CD73 inhibitor α,β- methylene ADP (left panel) or pan-adenosine receptor (AR) inhibitor CGS 15943 (right panel) was measured using a hemocytometer. The means +/- SEM from one representative of three experiments are shown and values significantly different from media control, determined by student’s t-test, are indicated by asterisk (*** = *p*< 0.0001; ** = *p*< 0.001; * = *p*<0.05). (B) Polarized H292 epithelial cells, pre-treated with increasing concentrations CD73 inhibitor α,β- methylene ADP (left panel) or just adenosine (right panel), were left uninfected (“media”) or infected apically with pneumococcus (“+ *S*. *pneumoniae*”). The transmigration of PMNs, added to the basolateral side in media alone or the indicated concentrations of CD73 inhibitor or adenosine was measured by myeloperoxidase ELISA. Data represent means +/- SEM, and shown are one of three separate experiments.

PMNs are recruited into the pulmonary airways via a multistep process involving first movement from the vasculature into the thin interstitial space and then across the lung epithelium into the airways. In several models, EAD limits the movement of PMNs across endothelial barriers [24,26]. To assess whether EAD targets the first step of pulmonary PMN recruitment, we measured the apical to basolateral movement of PMNs across monolayers of human umbilical vascular endothelial cells (HUVECS) grown on filter membranes in response to *S*. *pneumoniae* infection (see Methods; [39]). PMN migration was dependent on the infection of the endothelial cell monolayer by *S*. *pneumoniae* (Fig 4A), suggesting that, in this model, pneumococcal infection activates the endothelium to trigger PMN transmigration.

To test the role of EAD production on PMN migration in this system, we added the pharmacological inhibitor of CD73, α,β methylene ADP, to the media during the migration process, and found that it resulted in a significant dose-dependent increase in PMN migration in response to pneumococcal infection (Fig 4A, left panel). Importantly, a similar increase in PMN migration was observed when adenosine receptor signaling was blocked using the pan-adenosine receptor inhibitor CGS-15943 (Fig 4A, right panel).

We previously showed that blocking the movement of PMNs across the lung epithelium and into the airways protected mice against an otherwise lethal *S*. *pneumoniae* infection [9]. To test whether EAD also regulates PMN movement across this barrier, we utilized a well-established *in vitro* human PMN trans-epithelial migration assay [9]. As previously observed, apical pneumococcal infection of confluent polarized lung epithelial cells grown on filter membranes elicited robust basolateral to apical migration of PMNs (Fig 4B). Addition of the CD73 inhibitor, or exogenous adenosine to this assay had no significant effect on migration (Fig 4B). Together with our studies on endothelium, these results indicate that EAD negatively regulates PMN transmigration across endothelial but not epithelial monolayers in response to *S*. *pneumoniae* infection.

### Blocking EAD production or signaling increases interstitial pulmonary PMNs

**Fig 5:**
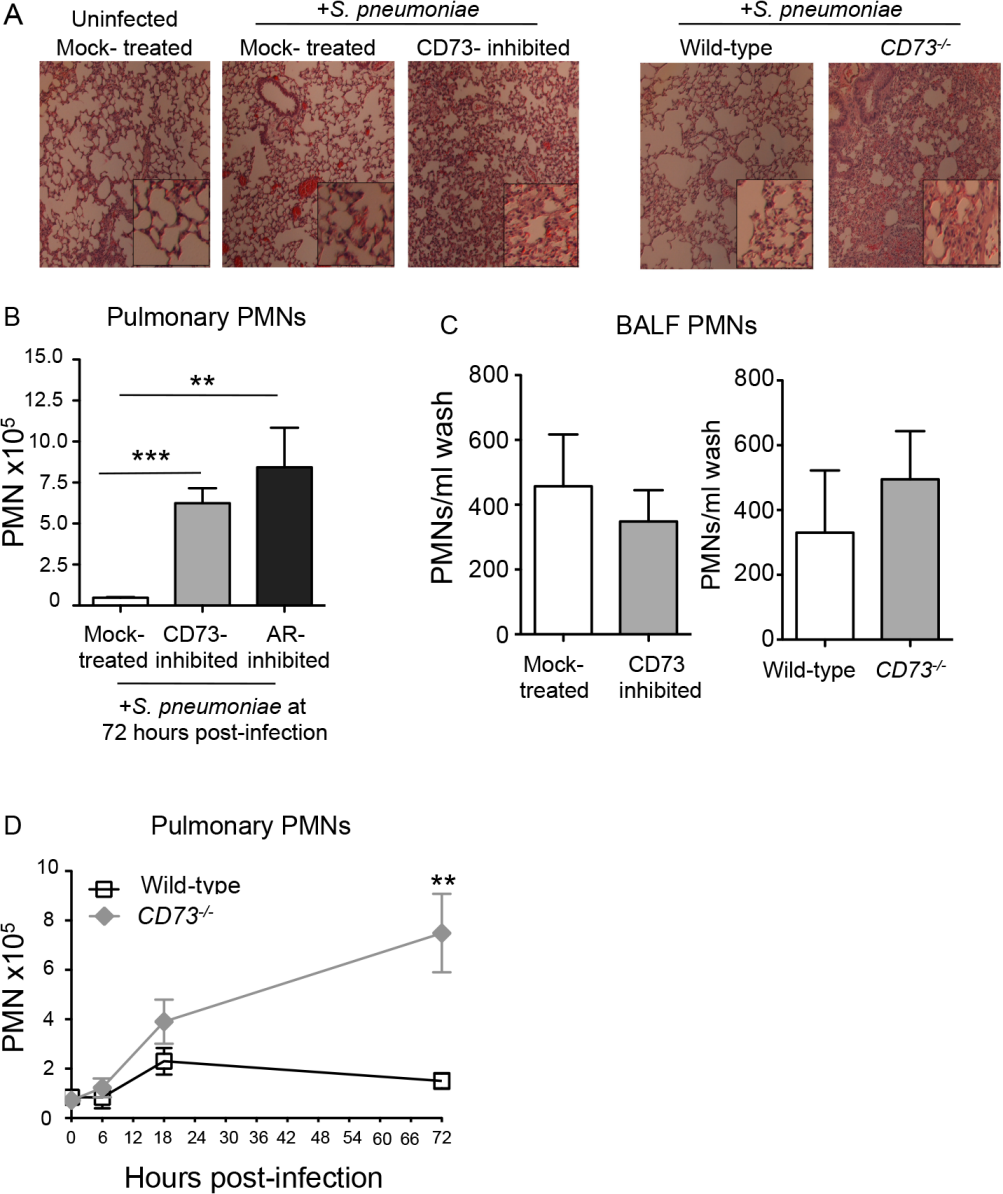
Inhibition of EAD production or signaling significantly increases PMN numbers in the pulmonary tissues. Wild-type untreated, mock-treated or CD73-inhibited C57BL/6 mice and CD73-ablated (*CD73* ^*-/-*^) mice were inoculated I.T. with 5x 10^3^ CFU of *S*. *pneumoniae* TIGR4. (A) H&E-stained lung sections examined by light microscopy at 3 days post-infection (10× magnification; inset at 40x magnification). (B) The mean +/- SEM of pulmonary PMNs (Ly6G^+^ cells) in mock-treated, CD73-inhibited, or adenosine receptor (AR)-inhibited mice, measured by flow cytometry at 72 hours post-infection. Uninfected mice of the different treatment groups had comparable low numbers (less than 10^5^) of PMNs in the lungs. (C) The number of PMNs in the bronchio-alveolar lavage fluid (BALF) was also determined by flow cytometry. (D) The mean +/- SEM of pulmonary PMNs of wild-type or *CD73* ^*-/-*^ mice was determined over time at the indicated times post-infection. Pooled data from three separate experiments (Panels B and C n = 8–9 mice per group; Panel D n = 6–9 mice per group) are shown. Statistically significant differences determined by student’s t-test are indicated by asterisks (*** = *p*< 0.0001; ** = *p*< 0.001; * = *p*<0.05).

To test whether EAD regulates transmigration specifically across pulmonary endothelium during infection, we administered 5x10^3^ CFU of *S*. *pneumoniae* I.T to mock-treated or CD73-inhibited mice, as well as to *CD73* ^*-/-*^ or wild type C57BL/6 mice. We assessed cellular recruitment into the lungs at day three post-infection, a time point that coincides with the resolution of pulmonary inflammation following a sub-lethal pneumococcal infection [40]. Consistent with the hypothesis that blocking EAD synthesis results in enhanced egress of PMNs from the vasculature, histological analysis of H&E stained lung sections at 3 days post-infection, revealed an increase in cellular infiltrates into the lungs of both *CD73* ^*-/-*^ mice and wild type C57BL/6 mice treated with the CD73 inhibitor (Fig 5A).

To quantify the apparent increase in pulmonary PMNs upon inhibition of EAD production or signaling, we measured pulmonary PMNs of mice that had been treated with the CD73 or the pan-adenosine receptor inhibitors and previously analyzed for lung and blood CFU in Fig 3A and 3B. Single-cell suspensions of lung tissue at day 3 post-infection were analyzed by flow cytometry after staining with antibody directed against the PMN marker Ly6G. Genetic ablation of CD73 (Fig 5D), as well as inhibition of CD73 or adenosine receptors (Fig 5B), resulted in a 6- to 8-fold increase in pulmonary PMNs, respectively, compared to mock-treated controls.

The failure of EAD to regulate PMN transmigration across human epithelial monolayers *in vitro* predicts that migration of PMNs into the airway spaces should be unaltered by manipulation of EAD signaling. To estimate the number of airway PMN, the number of PMNs in bronchoalveolar lavage fluid (BALF) of mock-treated and CD73-inhibited mice, or *CD73* ^*-/-*^ and wildtype control mice, three days after I.T. infection was determined by flow cytometry. In spite of the large increase in total pulmonary PMNs, no significant increase in the number of PMNs in BALF was observed upon pharmacological inhibition or genetic ablation of CD73 compared to control mice (Fig 5C). Importantly, the increase in pulmonary PMNs in the absence of CD73 was not simply a reflection of an increase in circulating PMNs, because both control and *CD73* ^*-/-*^ mice had comparable numbers of PMNs in the blood at 72 hours post-infection (S4 Fig). Our findings suggest that during pneumococcoal infection, EAD production and signaling are crucial for regulating the movement of PMNs specifically from the bloodstream across the endothelium, highlighting the differences in the regulation of PMN trafficking across the distinct endothelial and epithelial barriers.

To determine whether EAD production was important for regulating initial PMN recruitment, we assessed PMN influx into the lungs of *CD73* ^*-/-*^ and control mice infected I.T. with 5x10^3^ CFU of *S*. *pneumoniae* in both the early and later phases of infection in the set of mice previously analyzed for lung and blood CFU in Fig 3D. The two mouse strains displayed indistinguishable numbers of pulmonary PMNs at 6 hours post-infection, indicating the CD73-deficiency had no discernable effect on PMN influx into the lung in the first few hours of infection (Fig 5D). At 18 hours post-infection, the number of pulmonary PMNs in *CD73* ^*-/-*^ mice was 1.7-fold higher (*p* = 0.068) than in control in wild type mice. By 72 hours post-infection, PMN numbers returned to near-baseline levels in wild type mice but had increased two-fold in *CD73* ^*-/-*^ mice, reaching numbers ~four-fold higher compared to wild type (Fig 5D). These results suggest that EAD signaling has little or no effect on PMN recruitment early (i.e. 6 hours) after inoculation, but has a dramatic effect on PMN numbers 12 hours later (i.e. at 18 hours) and beyond, thereby interfering with the resolution of pulmonary inflammation during *S*. *pneumoniae* infection.

### EAD regulates the expression of several molecules critical for PMN transmigration during pulmonary challenge by *S*. *pneumoniae*

**Fig 6:**
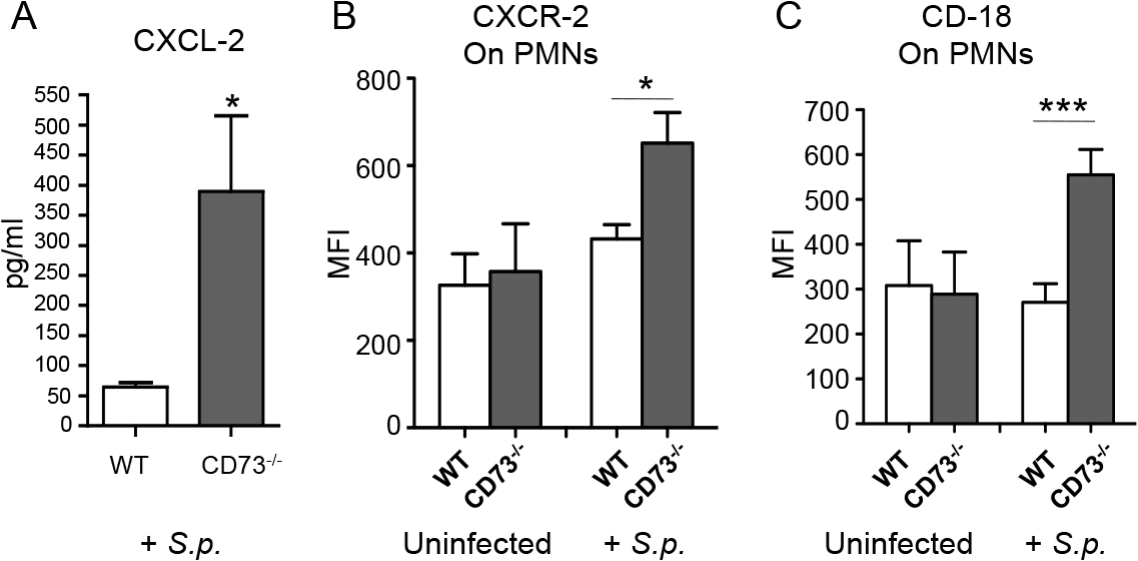
CD73 modulates the induction of leukocyte recruitment signals upon I.T. challenge by *S*. *pneumoniae*. Wild-type C57BL/6 or *CD73* ^*-/-*^ mice were mock-infected or I.T. challenged with 5 x 10^3^ CFU of *S*. *pneumoniae* TIGR4 (*+S*.*p*). Three days after challenge, levels of CXCL-2 in the lung homogenates were determined by ELISA (A) and the mean florescent intensities (MFI) of CXCR2 (B) or CD18 (C) on PMNs (Ly6G+) recruited into the lungs were determined by flow cytometry (see Materials and Methods). Pooled data from two separate experiments (n = 6 infected and n = 4 uninfected mice per group) are shown. Data represent means +/- SEM, and significant differences determined by Student’s t-test are indicated by asterisks (*** = *p*< 0.0001 and * = *p*<0.05).

PMN recruitment from the vasculature into the lung interstitial space involves a complex combination of chemotaxis signaling and cell adhesion molecule interactions [15,20]. In assessing whether EAD regulated some of the key molecules implicated in PMN recruitment into the lungs during pneumococcal infection, we found by ELISA that upon infection, *CD73* ^*-/-*^ mice had 7-fold higher levels of the chemokine CXCL2 in their lungs than did wild type mice (Fig 6A). Flow cytometric analysis revealed that the expression of the cognate receptor, CXCR2, was increased by 1.5-fold on the surface of PMNs from *CD73* ^*-/-*^ compared to wild type mice (Fig 6B). Similarly, levels of surface expressed β_2_ integrin (CD18), an adhesion molecule critical for PMN transmigration across endothelial barriers, was more than 2-fold higher on PMNs isolated from *CD73* ^*-/-*^ mice compared to wild type mice (Fig 6C). These findings suggest that EAD may be involved in regulating both chemotactic and cell adhesion steps during endothelial transmigration by PMNs.

### CD73-inhibition impairs the ability of PMNs to kill *S*. *pneumoniae* in vitro

**Fig 7:**
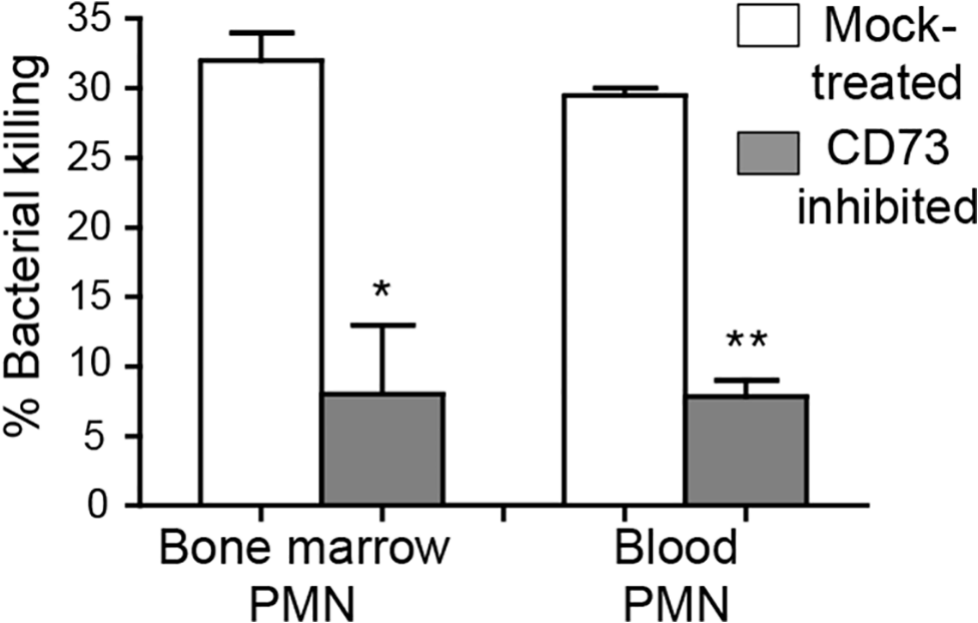
CD73 inhibition impairs the ability of PMNs to kill pneumococci. The viability of *S*. *pneumoniae* TIGR4 after 45 minute incubation with PMNs isolated from the blood and bone marrow of mock-treated C57BL/6 mice or mice treated with the CD73 inhibitor was determined by plating. The mean +/- SEM of % bacterial killing compared to a no PMN control was determined. Significant difference, determined by student’s t test, are indicated by asterisks (** = p<0.001 and * = p<0.05). Data representative of one of two separate experiments performed are shown (n = 4 mice per group).

Although CD73 inhibition resulted in enhanced recruitment of PMNs into the lungs, these PMNs failed to control infection. Indeed, by day 3 post-infection, CD73-inhibition was associated with ~100,000-fold more pulmonary pneumococci compared to mock-treatment (Fig 3). Thus, the absence of CD73 activity appeared to diminish the ability of PMNs recruited to the site of infection to clear the infection. We compared opsonophagocytic killing of pneumococci by PMNs isolated from the blood and bone marrow of CD73-inhibited or mock-treated mice. PMNs isolated from both the blood and bone marrow of CD73-inhibited mice displayed a ~5-fold defect in bacterial killing as compared to PMNs from mock-treated mice (Fig 7). These data are consistent with the suggestion that, in addition to a role for EAD in modulating transendothelial migration by PMNS, EAD may enhance bacteriocidal functions of PMNs.

### Depleting PMNs post-infection in the absence of EAD production restores resistance to pneumococcal challenge

**Fig 8:**
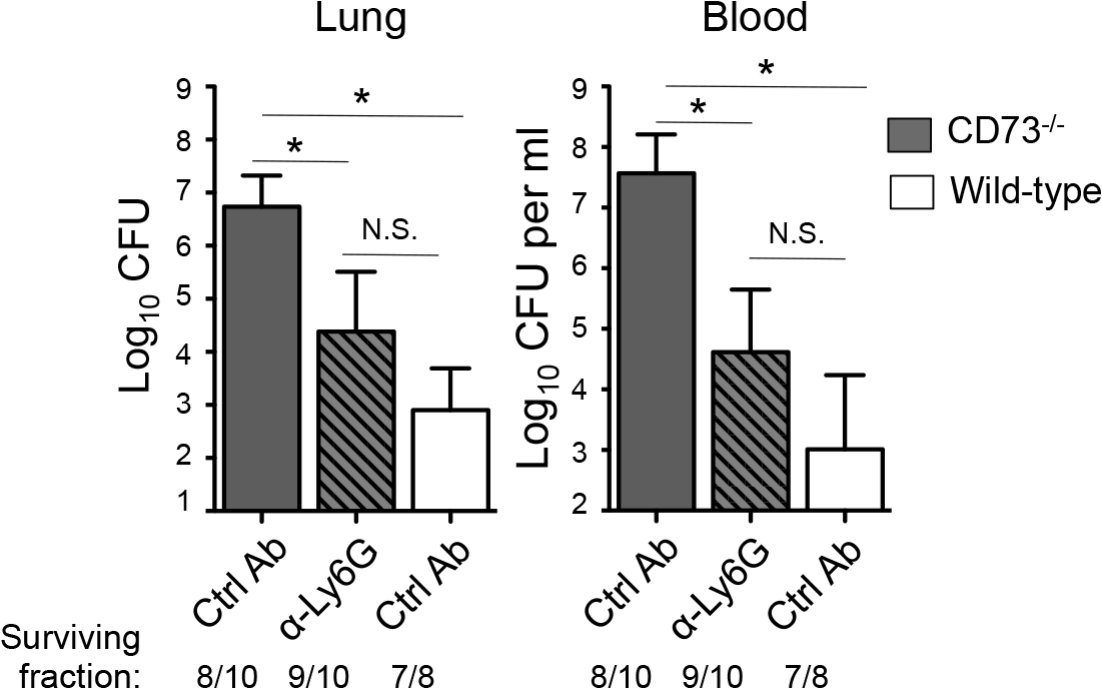
Post-infection depletion of PMNs partially reverses the susceptibility of *CD73* ^*-/-*^ mice to pneumococcal challenge. *CD73* ^*-/-*^ mice were treated with anti-Ly6G antibodies 18 hours after I.T inoculation of 5x10^3^ CFU of *S*. *pneumoniae* TIGR4. Three days after infection, bacterial burdens in the lungs and blood were compared to those of isotype control antibody-treated *CD73* ^*-/-*^ and wildtype mice. Pooled data from three separate experiments (n = 7 mice per group) are shown. Data represent means +/- SEM and significant (*p*<0.05) differences, determined by student’s t-test, are indicated by asterisk. Below the graphs are indicated the fraction of surviving mice within each group.

To determine whether the heightened *S*. *pneumoniae* susceptibility of mice inhibited for EAD signaling was due to a dysregulated recruitment and function of PMN, *CD73* ^*-/-*^ or wildtype control mice were treated with the Ly6G antibody 18 hours after I.T. infection with 5x10^3^ CFU of *S*. *pneumoniae* and bacterial burdens in the lungs, as well as spread to the blood were assessed. Treatment with the Ly6G antibodies resulted in ~80% PMN reduction in *CD73* ^*-/-*^ mice at day 3 post-infection compared to isotype-treated controls. In comparison to the untreated *CD73* ^*-/-*^ mice, PMN depleted *CD73* ^*-/-*^ mice had significantly lower bacterial burdens in both the lung and the blood, statistically indistinguishable (albeit slightly higher) from those of wildtype control mice (Fig 8). Our data suggest that the increased susceptibility of mice diminished for EAD signaling during pneumococcal infection is at least in part mediated by PMNs.

## Discussion

Acute inflammation following microbial infection may have either beneficial or detrimental effects. We investigated the role of PMNs in shaping the course of disease caused by the global pathogen *S*. *pneumoniae*. We first showed that within the first 12 hours after I.T. inoculation of mice, PMN entry into the lungs correlates with initial control of pulmonary bacterial burdens and that depletion of PMNs prior to pulmonary challenge with *S*. *pneumoniae* results in increased susceptibility and lethal septicemia. Although *S*. *pneumoniae* strains are quite heterogeneous, and PMN depletion enhances survival during murine infection by a serotype 8 pneumococcal strain [41], our current findings with strain TIGR4, a serotype 4 strain, are consistent with previous work indicating that PMNs, which phagocytose and kill pneumococci [42], are crucial for host defense against many serotypes of *S*. *pneumoniae* [13–15,43].

We also found that in the next phase of infection, beginning at approximately twelve hours after inoculation, PMN influx into the lungs corresponded with increased bacterial lung burdens and pneumoccocal spread into the systemic circulation. Depletion of PMNs 18 h after pulmonary challenge resulted in lower bacterial loads and enhanced survival, suggesting that timely resolution of inflammation may diminish deleterious effects of an over-exuberant host response. Indeed, mice that survived infection had drastically fewer pulmonary PMNs at day 3 post-infection, and many studies have shown that conditions that result in increased numbers of PMNs in the lungs several days after *S*. *pneumoniae* lung infection, such as influenza virus infection [18], aging [8,16,44] or deficiency in regulatory T cells [17], suffer more severe systemic spread and reduced survival. Conversely, reducing chemotaxis of PMNs into airways after I.T. pneumococcal challenge of mice resulted in uniform survival after an otherwise lethal pneumococcal pulmonary challenge [9]. Mice protected from *S*. *pneumoniae* challenge by treatment with anti-capsular antibody experience only transient influx in PMNs into the lung followed by resolution by 24 hours post-infection [45]. Thus, although PMNs are initially needed to clear *S*. *pneumoniae* infection, later in infection they function in ways that are detrimental to the host, suggesting that regulation of PMN influx is crucial to protect against disease.

Although EAD is a crucial regulator of acute pulmonary inflammation in several sterile lung injury models [24,25,46,47], its role in infection-induced inflammation remains relatively unexplored. EAD is recognized by four distinct adenosine receptors, termed A1, A2A, A2B and A3, and stimulation of a particular adenosine receptor may have a positive or negative effect on pulmonary inflammation depending on the type of lung injury [23]. A1 receptor stimulation diminished PMN infiltration and tissue damage in murine lung injury models [48–50] but promoted damaging lung inflammation during influenza infection [29]. Stimulation of the A2B adenosine receptor blocked LPS-mediated PMN recruitment into the lungs in mice [46,47,51], but had no effect on leukocyte recruitment following pulmonary infection by the Gram-negative bacterium *K*. *pneumoniae* [28]. In the context of the Gram-positive pathogen, *S*. *pneumoniae*, we found here that EAD negatively regulates trans-endothelial migration *in vitro*, and inhibition of EAD signaling by pan-adenosine receptor blockade, or by genetic ablation or chemical inhibition of CD73, resulted in a four- to 20-fold fold increase in pulmonary PMNs three days following I.T. pneumococcal challenge. Adenosine enhanced basolateral-to-apical transmigration of PMNs across endothelial monolayers *in vitro*, but did not regulate PMN migration across epithelial monolayers. Correspondingly, the increase in pulmonary PMNs during murine infection was not reflected in an increase in airway PMNs, as sampled by bronchoalveolar lavage. Thus, similar to previous findings after A2B receptor inhibition in an LPS-induced lung injury model [51], upon disruption of EAD signaling, PMNs accumulated predominantly in the interstitium.

The mechanism by which EAD modulates PMN transendothelial migration during pneumococcal infection could involve chemotactic signals or molecules that directly mediate PMN-endothelial cell interactions, or both. *In vitro*, the production of the chemokine CXCL-8 (IL-8) by endothelial monolayers is diminished by adenosine [52], and in a murine LPS-induced lung injury model, the level of CXCL2/3 (i.e. the murine paralog of IL-8), a chemokine that promotes PMN and macrophage recruitment during murine pneumococcal infection [20], is diminished by A1 receptor stimulation [48]. On activated PMNs, adenosine inhibits up-regulation of the β2 integrin CD11b/CD18 [53], which has been implicated in pulmonary PMN recruitment during pneumococcal murine infection[15]. We found that, following pneumococcal infection, the level of pulmonary CXCL-2 was significantly elevated in *CD73* ^*-/-*^ mice compared to wild-type mice. In addition, levels of CXCR-2 (i.e. the CXCL-2 receptor) and the integrin CD18 were elevated on *CD73* ^*-/-*^ PMNs. Thus, EAD likely regulates multiple signals involved in pulmonary recruitment of PMNs in response to *S*. *pneumoniae* infection.

A striking finding was that disruption of EAD production or signaling resulted in an increase of many orders of magnitude in bacterial numbers in the lung and blood, as well as significantly higher mortality rates. Conversely, inhibition of EAD breakdown decreased bacterial loads and diminished lethality. Although we cannot rule out that altering extracellular ATP or adenosine levels in the host may have direct effects on *S*. *pneumoniae*, especially given their far ranging metabolic and/or regulatory effects on pneumococcus [5], neither adenosine nor the ADA or CD73 inhibitors altered *S*. *pneumoniae* viability *in vitro*. EAD can regulate PMN phagocytosis and degranulation *in vitro* [22], features that are crucial for anti-pneumococcal activity of PMNs [42]. Interestingly, several Gram-positive pathogens (although likely not *S*. *pneumoniae*) express ectonucleotidases that produce EAD that inhibits PMN-mediated phagocytosis [54] and oxidative killing [55]. A2B-deficient PMNs form neutrophil extracellular traps (NETs) and clear *K*. *pneumoniae* more efficiently than wild type PMNs [28]. In contrast, here we found that pharmacologic blockade of CD73 impaired opsonophagocytic killing of *S*. *pneumoniae* by PMNs *ex vivo*. Phagocytic killing of pneumococci by PMNs requires serine proteases but is independent of oxidative burst [13,42], raising the possibility that the effect of EAD on a given infection may depend on the specific mechanism(s) by which PMNs kill the particular infecting microbe.

Importantly, however, the effects of CD73 ablation or inhibition and adenosine signaling blockade on lung infection cannot be fully explained by the loss of a putative PMN defense function, because depletion of these cells 18 hours after inoculation significantly mitigated the susceptibility of *CD73* ^*-/-*^ mice. Thus, EAD appears to limit disease by blunting the detrimental effect of PMNs later in infection. The nature of this PMN-mediated harmful effect on immune control is unknown, but it is possible that once bacterial burden reaches a threshold beyond which PMNs can no longer control the infection, they instead contribute to an environment permissive for bacterial persistence and growth. Some pathogens, such as *Salmonella enterica*, harbor metabolic capacities well adapted to the inflamed environment [56], and given that sugar utilization and other metabolic pathways have been shown to be critical determinants of pneumococcal virulence *in vivo* [57], PMN-derived products in inflamed tissue might make available growth-limiting nutrients utilized by this organism [58]. PMNs are also known to modulate other arms of the host immune response, such as the recruitment and function of T cells [59] and monocytes [60], and may influence pneumococcal persistence indirectly. Finally, although PMN depletion following infection significantly mitigated the susceptibility of *CD73* ^*-/-*^ mice, these mice still suffered somewhat (albeit not statistical significant) higher bacterial burdens than wild-type mice. Thus, EAD, which regulates the function of immune cells such as macrophages [61] and regulatory T- cells [62,63] that promote pneumococcal defense [17,64], may also enhance resistance by PMN-independent mechanisms.

In addition to reducing bacterial burden in the lungs, we found a strong correlation between pulmonary inflammation and systemic spread. Inhibition of EAD production or receptor signaling resulted in high levels of both pulmonary inflammation and bacteremia, whereas PMN depletion 18 hours post-infection or chemical inhibition of adenosine breakdown reduced bacterial spread. Although the reduced spread may partially reflect lower bacterial burden in the lung, CD73-inhibited mice challenged with a high (10^7^) dose of pneumococci harbored numbers of bacteria in the lung equivalent to untreated controls, yet suffered greater bloodstream spread. In other infection models, PMN influx into infected tissues was associated with tissue damage and poor infection outcome, without altering pathogen numbers [65]. In addition, we previously showed that transmigration of PMN across a respiratory epithelial monolayer disrupted its barrier function *in vitro* and inhibition of PMN influx into the airways prevented lethal septicemia in mice [9]. Given that we found here that EAD signaling controls transmigration across endothelium but not epithelium, inflammation may promote disseminated pneumococcal disease by multiple mechanisms.

All four EAD receptors are produced in the lung [21] and on PMNs [22], and in future studies it will be essential to characterize the adenosine receptor(s) that influence the course of pneumococcal infection. Adenosine receptors vary in both their effect on pulmonary inflammation and their affinity for adenosine, with EC_50_‘s varying from <0.5 to 64μM, raising the possibility that EAD could be pro- or anti-inflammatory depending on EAD tissue concentration. Previous studies indicate that administration of the ADA inhibitor EHNA-hydrochloride and the CD73 inhibitor α,β methylene ADP to mice results in the predicted effects on adenosine [24,33] concentration, but we did not directly measure changes in EAD levels during pneumococcal infection. Changes in the expression of adenosine receptors [61] could also raise another dynamic variable that may influence EAD signaling. Adenosine receptor signaling resulted in either a pro-, or anti-inflammatory T-cell response during autoimmune uveitis depending on the phase of the disease [66], and one might imagine that the effect of EAD signaling may differ with phase of pneumococcal infection, providing a rationale for the lack of discernable effect of CD73 inhibition or ablation soon after I.T. inoculation, but a dramatic effect on the resolution of pulmonary inflammation later in infection. The use of receptor-specific agonists and antagonist or mice that are genetically ablated for a specific adenosine receptor provide future avenues to better define specific pathways that control inflammation and disease during pneumococcal infection, potentially revealing new therapeutic strategies to combat this important disease.

## Material and Methods

### Ethics statement

This work was performed in accordance with the recommendations in the Guide for the Care and Use of Laboratory Animals published by the National Institutes of Health. All procedures were reviewed and approved by Tufts University Institutional Animal Care and Use Committee (IACUC) and are under protocol # B2014-86.

### Mice

Wild type BALB/c/By/J (BALB/c), C57BL/6J (B6) and CD73 knockout (CD73^-/-^) mice on a B6 background were purchased from The Jackson Laboratory (Bar Harbor, ME) and bred at Tufts University. Mice were matched for age and sex and maintained in a specific-pathogen free facility at Tufts University.

### Murine infections

Mice were challenged I.T. with *S*. *pneumoniae* TIGR4 grown at 37°C in 5% CO_2_ in Todd-Hewitt broth (BD Biosciences) supplemented with 0.5% yeast extract (THY) and oxyrase as previously described [9]. For every experiment, the inoculum was plated on blood agar plates for CFU enumeration. If the bacterial inoculae differed by less than 20%, the data from separate experiments were pooled. If the titers varied by more 20% between individual experiments, then representative data are shown.

### EAD-pathway inhibitors

The effect of EAD on infection was assessed using the following: The selective and competitive inhibitor of CD73, α,β methylene ADP; the pan-adenosine receptor antagonist CGS-15943; and the adenosine deaminase inhibitor EHNA hydrochloride. All chemicals were purchased from Sigma Aldrich, dissolved in DMSO and filter sterilized by passing through a 0.22μm filter. The mice were then given intraperitoneal (i.p.) injections of 10mg/kg daily at days 0 (immediately before I.T. infection), 1 and 2 post-infection. Control mice were mock-treated with the vehicle control.

### Assessment of bacterial burden and survival

For enumeration of bacterial numbers, lung and blood samples were harvested from the live mice and plated on TSA plates supplemented with 5% sheep blood agar (Northeast Laboratory Services). The limit of detection was 20 CFU (1.3 Log_10_) per lung and 200 CFU (2.3 Log_10_) per ml blood. When no colonies were detected on the plates, the numbers of bacteria were assumed to be slightly under our limit of detection (2.0 Log_10_ bacteria per 1ml of blood and 1.0 Log_10_ bacteria in the lungs). For survival analysis, the mice were monitored for 7 days following infection. At a dose of 5x10^5^ CFU *S*. *pneumoniae* TIGR4, we typically observed ~ 60% total mortality rate over the course of a week, with all deaths occurring on days 2, 3 or 4. However, the specific kinetics of death over this three-day period varied between experiments. We consistently observed an ~70% survival rate in mice inoculated with 5x10^3^ CFU of *S*. *pneumoniae* TIGR4. Despite slight variation in kinetics between experiments, the differences between experimental groups were consistent from experiment to experiment.

### Bacterial growth assays

Frozen aliquots of log-phase *S*. *pneumoniae* TIGR4 strain (serotype 4) were thawed, washed and diluted to an OD_600_ ~ 0.1 in THY liquid media supplemented with oxyrase. To measure the effect of the CD73 inhibitor on bacterial growth, 40μg/ml of the drug was added to the media. To measure the effect of adenosine on growth, the chemical (Sigma Aldrich) was added exogenously to a final concentration of 10μM or 100μM. The compounds were added at 0 h and bacterial growth in 37°C / 5% CO_2_ was monitored overtime by measuring OD_600_ and compared to growth in media alone. To measure the effect of the adenosine deaminase inhibitor and the pan-adenosine receptor inhibitor on bacterial viability, 40μg/ml of the drugs was added to the media at 37°C / 5% CO_2_ and two hours later bacterial viability was measured by plating on blood agar plates for CFU enumeration.

### PMN depletions

Mice were injected i.p. with 100 μg of the Ly6G-depleting antibody IA8 or isotype IgG control (BD Bioscience). For preinfection depletion, mice received one injection per day at 24 hours pre-infection, the time of infection plus 18 and 48 hours post-infection. For depletion 18 hours post-infection,mice were given one injection per day at 18 and 48 hours post-infection. Treatments resulted in depletion of >90% in wildtype mice and ~80% in *CD73* ^*-/-*^ mice of circulating and lung PMNs at day 3 post-infection as compared to isotype-treated controls.

### Isolation of cells from alveolar spaces and lung tissues

Mice were euthanized at the indicated times post-infection and the bronchio-alveolar lavage fluid (BALF) obtained by washing the lungs with PBS. The lungs were then digested with Type II collagenase (Worthington) and DNase (Worthington) and single-cell suspensions obtained as previously described [8].

### Flow cytometry

Cells were stained with anti-mouse Ly6G (clone 1A8, BD Biosciences), CD18 (Clone M18/2, Biolegend) and CXCR2 (Clone SA045E1, Biolegend) antibodies. Fluorescence intensities were measured on a FACSCalibur and at least 25,000 events for lung tissue and 10,000 events for BALF were analyzed using FlowJo.

### CXCL-2 ELISA

Three days post-infection, the lungs were harvested, homogenized in sterile PBS and the resulting supernatants were used CXCL-2 concentrations using the mouse MIP2/CXCL-2 ELISA kit (Sigma-Aldrich) following the manufacturer’s protocol.

### Histology

For histological analysis mice were euthanized 3 days post-infection and whole lungs were fixed in 10% buffered formalin (Sigma-Aldrich). Lungs were then embedded in paraffin, sectioned at 5μm, stained with hematoxylin and eosin (H&E) and analyzed using a Nikon eclipse TE2000-U microscope.

### Isolation of human PMNs

Healthy human volunteers were recruited in accordance to IRB protocols and signed informed consent forms. Whole blood was obtained and anticoagulated with acid citrate/dextrose. PMNs were isolated using a 2% gelatin sedimentation technique as previously described [9].

### Maintenance of epithelial cells

Human pulmonary mucoepidermoid carcinoma-derived NCI-H292 (H292) cells were grown on the underside of collagen-coated Transwell filters (0.33-cm^2^, Corning Life Sciences) in RPMI 1640 medium (ATCC) with 2 mM L-glutamine, 10% FBS, and 100 U penicillin/streptomycin following a previously described protocol [9].

### PMN migration assay across epithelium

Transmigration assay was performed as previously described [9] with pneumococcal infection and PMN migration time of 2.5 h. When indicated, the migration was allowed to occur in HBSS +/- EAD, the CD73 or adenosine receptors inhibitor at the indicated concentrations. PMNs that transmigrated into the apical chamber were measured by the myeloperoxidase ELISA following a well established assay [67] after their collection and lysis in 10% Triton-X 100 to release the myeloperoxidase. Myeloperoxidase ELISA of serial dilutions of known numbers of neutrophils were used to establish a standard curve, which was then used to quantitated migrated neutrophils.

### Maintenance of endothelial cells

Human umbilical vascular endothelial cells (HUVECs) were seeded on the inner chamber of collagen-coated Transwell filters (0.33-cm^2^, Corning Life Sciences) in M199 medium (Biowhittaker) supplemented with 2 mM L-glutamine, 10% FBS, 10μg/ml endothelium mitogen (Fisher), 20μg/ml heparin sodium salt (Sigma) and 100 U penicillin/streptomycin following a previously described protocol [68]. The cell monolayer was allowed to form over 4–5 days.

### PMN migration assay across endothelium

The PMN migration assay across the endothelium was performed as previously described [39] with the following modifications. The endothelial cells seeded on Transwells were infected for 3 h by *S*. *pneumoniae* added to the lower chamber. 5x10^5^ PMNs were added to the upper chamber and migration was allowed to occur +/- CD73 or adenosine receptors inhibitors for 3 h at 37°C/ 5% CO_2_. Since this assay utilizes standard RPMI with phenol red, which precludes colorimetric assays such as MPO, the number of PMNs that migrated was determined by counting in a hemacytometer in triplicate, as previously described [39]. The inhibitors had no significant affect on cell viability within the timeframe of the assay as measured by trypan-blue exclusion.

### Isolation of murine PMNs

Bone marrow PMNs were isolated from the femurs of mice as previously described [43] and enriched using Percoll (Sigma) density gradient centrifugation. For isolation of PMNs from the circulation, blood was collected by cardiac puncture using acid citrate/dextrose as an anticoagulant. PMNs were then enriched by Ficoll density gradient centrifugation in Mono-poly (MP-Biomedicals) resolving medium based on the manufacturer’s instructions. The enriched cells were ~ 85–90% Ly6G^+^ by flowcytometry.

### Opsonophagocytic killing assay

The ability of PMNs to kill pneumococci was assessed *ex vivo* as previously described. Briefly, 200μl reactions in Hank’s buffer/0.1% gelatin consisted of 1x10^5^ PMNs incubated with 1x10^2^ bacteria grown to mid log phase and pre-opsonized with 20μl mouse sera. The reactions were incubated rotating for 45 minutes at 37°C. Percent killing relative to parallel incubations without PMNs was determined by plating serial dilutions on blood agar plates.

### Statistics

All statistical analysis was performed using Prism4 for Macintosh (Graph Pad). For analysis of survival curves, Log-rank (Mantel-Cox) test was performed. CFU data were log-transformed to normalize distribution. Student t-test was used for comparison between groups. *p* values less than 0.05 were considered significant. For all graphs, the mean values +/- SEM are shown.

## Supporting Information

S1 Fig Extracellular adenosine and the CD73 inhibitor do not affect bacterial growth *in vitro*. Growth of *S*. *pneumoniae* TIGR4 was measured in THY media after the addition of (A) increasing concentrations of exogenously added adenosine or (B) the CD73 inhibitor (40μg/ml). (C) Viability of *S*. *pneumoniae* TIGR4 was measured after 2 h incubation in THY media +/- the addition of the ADA-inhibitor EHNA-hydrochloride or the adenosine receptors (AR)-inhibitor CGS 15943 (40μg/ml). Representative data from (A) three and (B and C) two separate experiments are shown.
(TIF)

S2 Fig Adenosine mediated-protection against *S*. *pneumoniae* lung infection is in part dependent on adenosine receptors. Wild type C57BL/6 mice were either given the adenosine deaminase (ADA) inhibitor (EHNA hydrochloride) alone or in conjunction with a pan adenosine receptor (AR) inhibitor (CGS 15943). Survival (D) as well as bacterial numbers in the lung (A) and blood (B) at 3 days post lung infection with 5x10^5^ CFU of *S*. *pneumoniae* TIGR4 were assessed. Representative data from one of two separate experiments are shown. ** = *p*< 0.001; * = *p*<0.05 indicate that the means are significantly different by student’s t-test.
(TIF)

S3 Fig Inhibition of EAD production increases systemic spread of the pneumococci following high dose lung challenge. Mock-treated and CD73-inhibited BALB/c mice were challenged I.T. with ~1x10^6^ CFU of *S*. *pneumoniae* TIGR4. Bacteremia was monitored overtime. Data represent means +/- SEM. Significant (*p*<0.05) differences are indicated by asterisk. Pooled data from two separate experiments (n = 5 mice per group) are shown. None of the mice succumbed to infection within the monitored time.
(TIF)

S4 Fig The effect of CD73 ablation on immune cell numbers in the blood Wildtype control and *CD73* ^*-/-*^ mice were either inoculated intratracheally with 5× 10^3^ CFU of *S*. *pneumoniae* TIGR4 (+*Sp*) or left uninfected. At day 3 post-infection, the number of PMNs (Ly6G^+^) that were circulating in the blood was determined by flow cytometry. Data shown are pooled from three separate experiments performed (n = 9 mice per group). * = p<0.05 indicate that the means are significantly different by student’s t-test.
(TIF)
