# Supplementary figures and images for: Extracellular Adenosine Protects against Streptococcus pneumoniae Lung Infection by Regulating Pulmonary Neutrophil Recruitment
Source: PLoS Pathog. 2015 Aug 27;11(8):e1005126. doi: 10.1371/journal.ppat.1005126 (PMC4552087; doi:10.1371/journal.ppat.1005126)

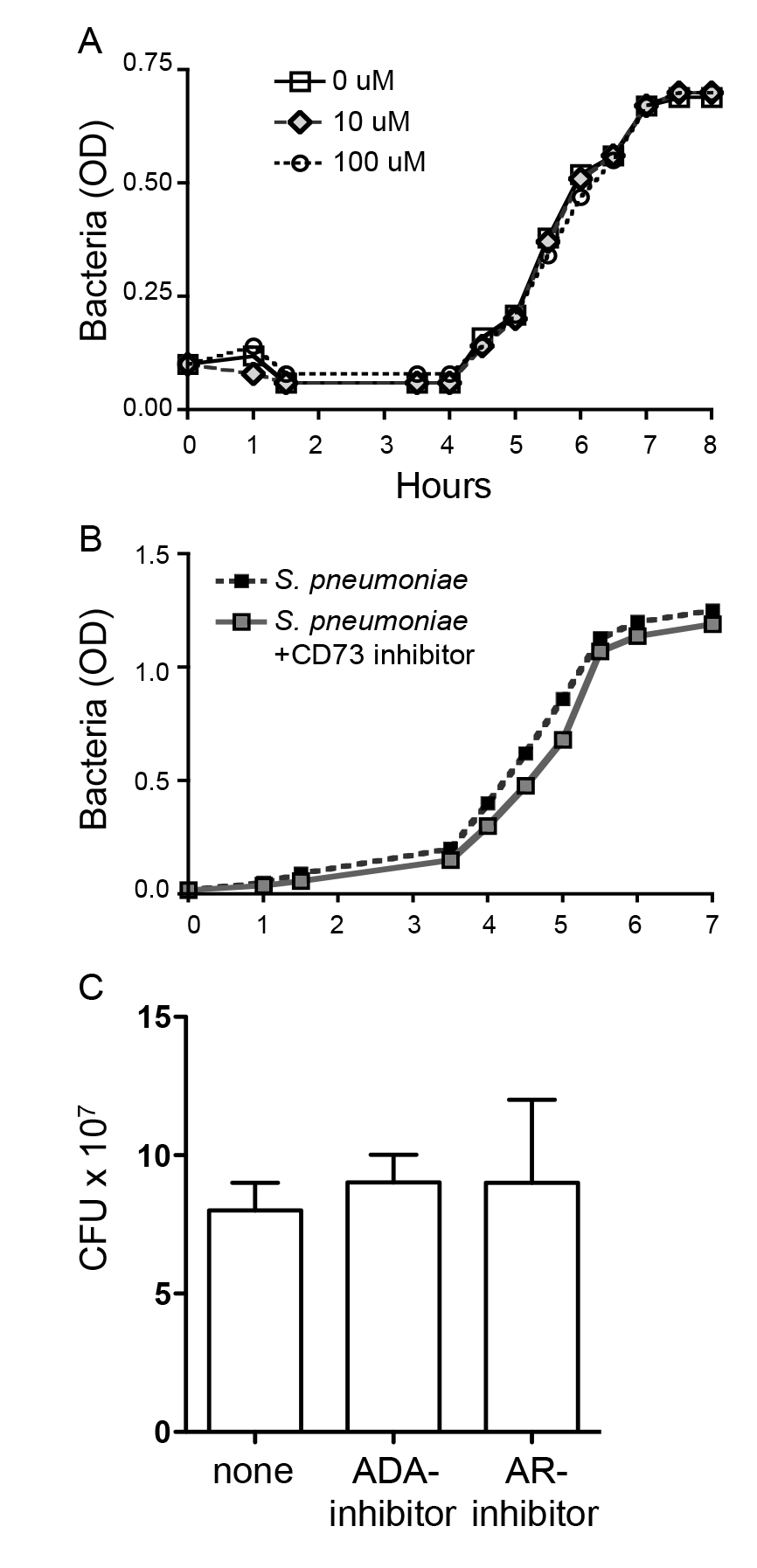

Supplement: S1 Fig — Growth of S. pneumoniae TIGR4 was measured in THY media after the addition of (A) increasing concentrations of exogenously added adenosine or (B) the CD73 inhibitor (40μg/ml). (C) Viability of S. pneumoniae TIGR4 was measured after 2 h incubation in THY media +/- the addition of the ADA-inhibitor EHNA-hydrochloride or the adenosine receptors (AR)-inhibitor CGS 15943 (40μg/ml). Representative data from (A) three and (B and C) two separate experiments are shown. (TIF) [file ppat.1005126.s001.tif]

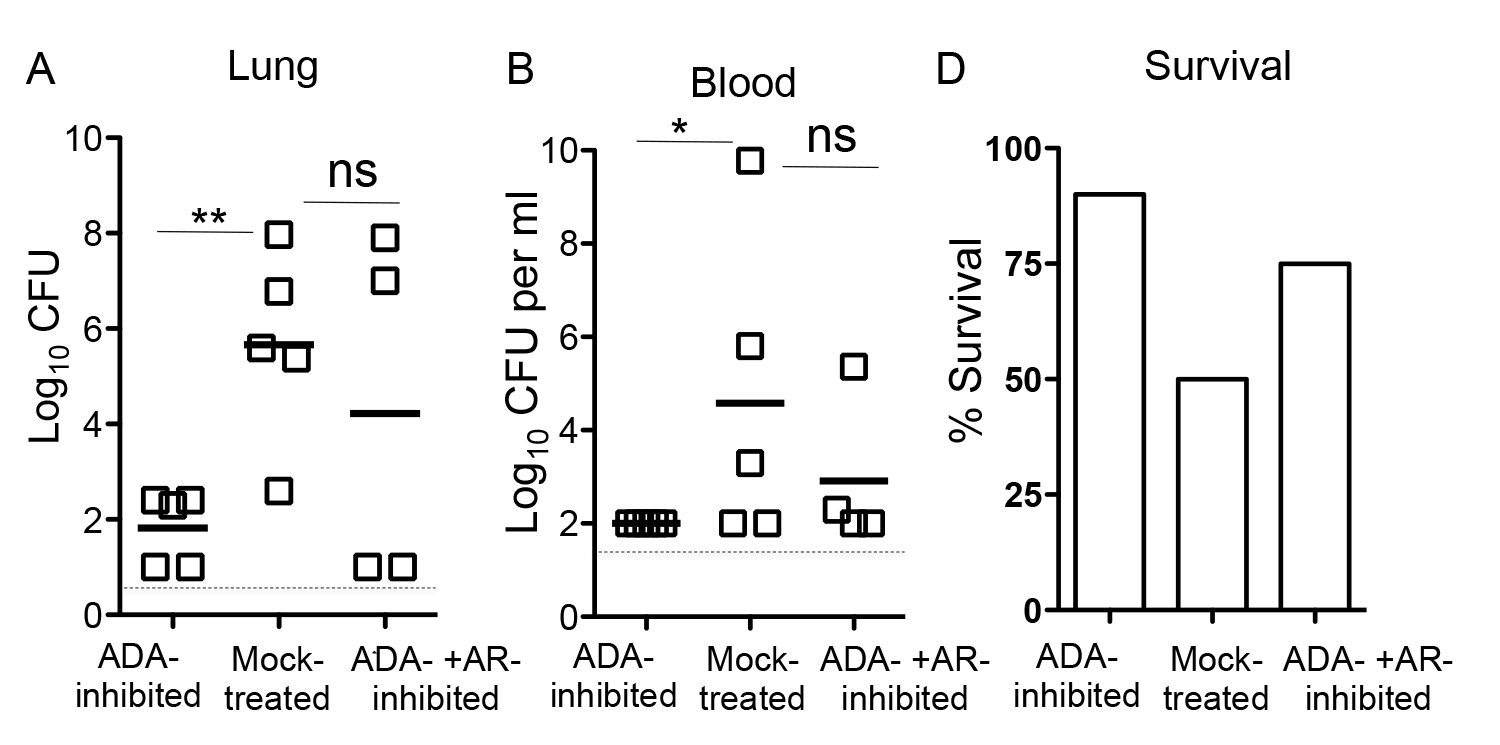

Supplement: S2 Fig — Wild type C57BL/6 mice were either given the adenosine deaminase (ADA) inhibitor (EHNA hydrochloride) alone or in conjunction with a pan adenosine receptor (AR) inhibitor (CGS 15943). Survival (D) as well as bacterial numbers in the lung (A) and blood (B) at 3 days post lung infection with 5x10^5^ CFU of S. pneumoniae TIGR4 were assessed. Representative data from one of two separate experiments are shown. ** = p< 0.001; * = p<0.05 indicate that the means are significantly different by student’s t-test. (TIF) [file ppat.1005126.s002.tif]

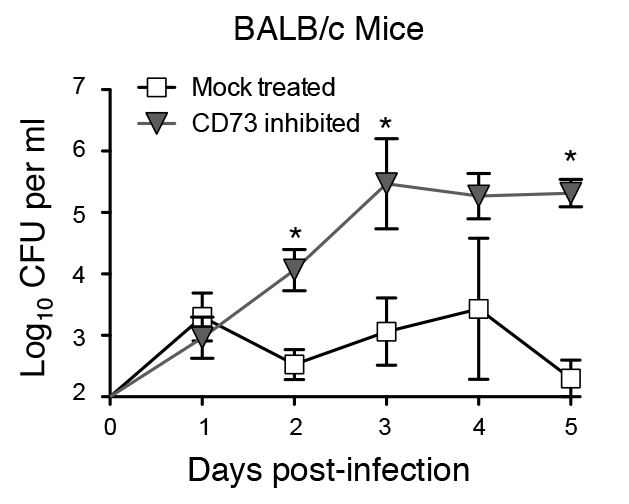

Supplement: S3 Fig — Mock-treated and CD73-inhibited BALB/c mice were challenged I.T. with ~1x10^6^ CFU of S. pneumoniae TIGR4. Bacteremia was monitored overtime. Data represent means +/- SEM. Significant (p<0.05) differences are indicated by asterisk. Pooled data from two separate experiments (n = 5 mice per group) are shown. None of the mice succumbed to infection within the monitored time. (TIF) [file ppat.1005126.s003.tif]

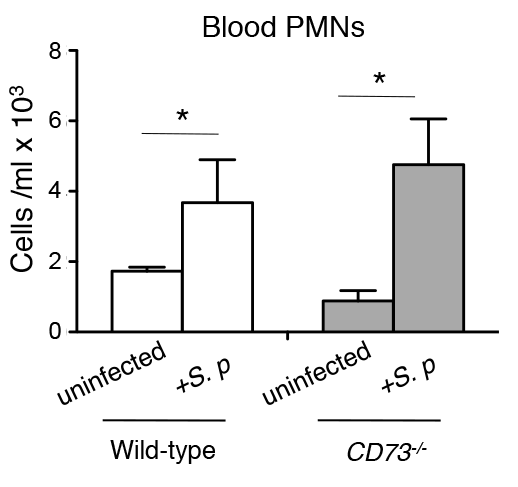

Supplement: S4 Fig — At day 3 post-infection, the number of PMNs (Ly6G+) that were circulating in the blood was determined by flow cytometry. Data shown are pooled from three separate experiments performed (n = 9 mice per group). * = p<0.05 indicate that the means are significantly different by student’s t-test. (TIF) [file ppat.1005126.s004.tif]
